# Identification of two novel pathogenic variants of *PIBF1* by whole exome sequencing in a 2-year-old boy with Joubert syndrome

**DOI:** 10.1186/s12881-020-01130-x

**Published:** 2020-10-01

**Authors:** Yue Shen, Hao Wang, Zhimin Liu, Minna Luo, Siyu Ma, Chao Lu, Zongfu Cao, Yufei Yu, Ruikun Cai, Cuixia Chen, Qian Li, Huafang Gao, Yun Peng, Baoping Xu, Xu Ma

**Affiliations:** 1 National Research Institute for Family Planning, Beijing, China; 2 National Human Genetic Resources Center, Beijing, China; 3 China National Clinical Research Center of Respiratory Diseases, Respiratory Department of Beijing Children’s Hospital, Capital Medical University, National Center for Children’s Health, Beijing, China; 4 Department of Radiology, Beijing Children’s Hospital, Capital Medical University, National Center for Children’s Health, Beijing, China

**Keywords:** *PIBF1*, Joubert syndrome, Cerebellar vermis hypoplasia, Whole exome sequencing

## Abstract

**Background:**

Joubert syndrome (OMIM 213300) is an autosomal recessive disorder with gene heterogeneity. Causal genes and their variants have been identified by sequencing or other technologies for Joubert syndrome subtypes.

**Case presentation:**

A two-year-old boy was diagnosed with Joubert syndrome by global development delay and molar tooth sign of mid-brain. Whole exome sequencing was performed to detect the causative gene variants in this individual, and the candidate pathogenic variants were verified by Sanger sequencing. We identified two pathogenic variants (NM_006346.2: c.1147delC and c.1054A > G) of *PIBF1* in this Joubert syndrome individual, which is consistent with the mode of autosomal recessive inheritance.

**Conclusion:**

In this study, we identified two novel pathogenic variants in *PIBF1* in a Joubert syndrome individual using whole exome sequencing, thereby expanding the *PIBF1* pathogenic variant spectrum of Joubert syndrome.

## Background

Joubert syndrome (OMIM: 213300) is an autosomal recessive disorder characterized by a specific mid-hindbrain malformation, hypotonia and developmental delay/intellectual impairment [1]. Molar tooth sign of mid-brain is a diagnostic standard for Joubert syndrome [2]. As Joubert syndrome is a genetically heterogeneous disease, causal genes and their variants have been identified with improved sequencing technologies. Individuals with Joubert syndrome have many of the clinical features of ciliopathies [3], and many ciliary/basal body genes and variants have been discovered to be associated with Joubert syndrome [4, 5]. Joubert syndrome 33 (JBTS33) is caused by *PIBF1*. Wheway et al. first identified 4 variants or deletions in the *PIBF1* gene associated with Joubert syndrome from Hutterite families and other families [6]. A homozygous 36-bp insertion in *PIBF1* (c.1181_1182ins36) in a Joubert syndrome family has been reported by Hebbar M. et al. [7]. Moreover, Ott T. et al. found a compound heterozygote (c.1453C > T and c.1508A > G) in a German patient [8].

In this study, we identified two novel pathogenic variants on *PIBF1* in a Joubert syndrome individual using whole exome sequencing.

## Case presentation

**Fig. 1:**
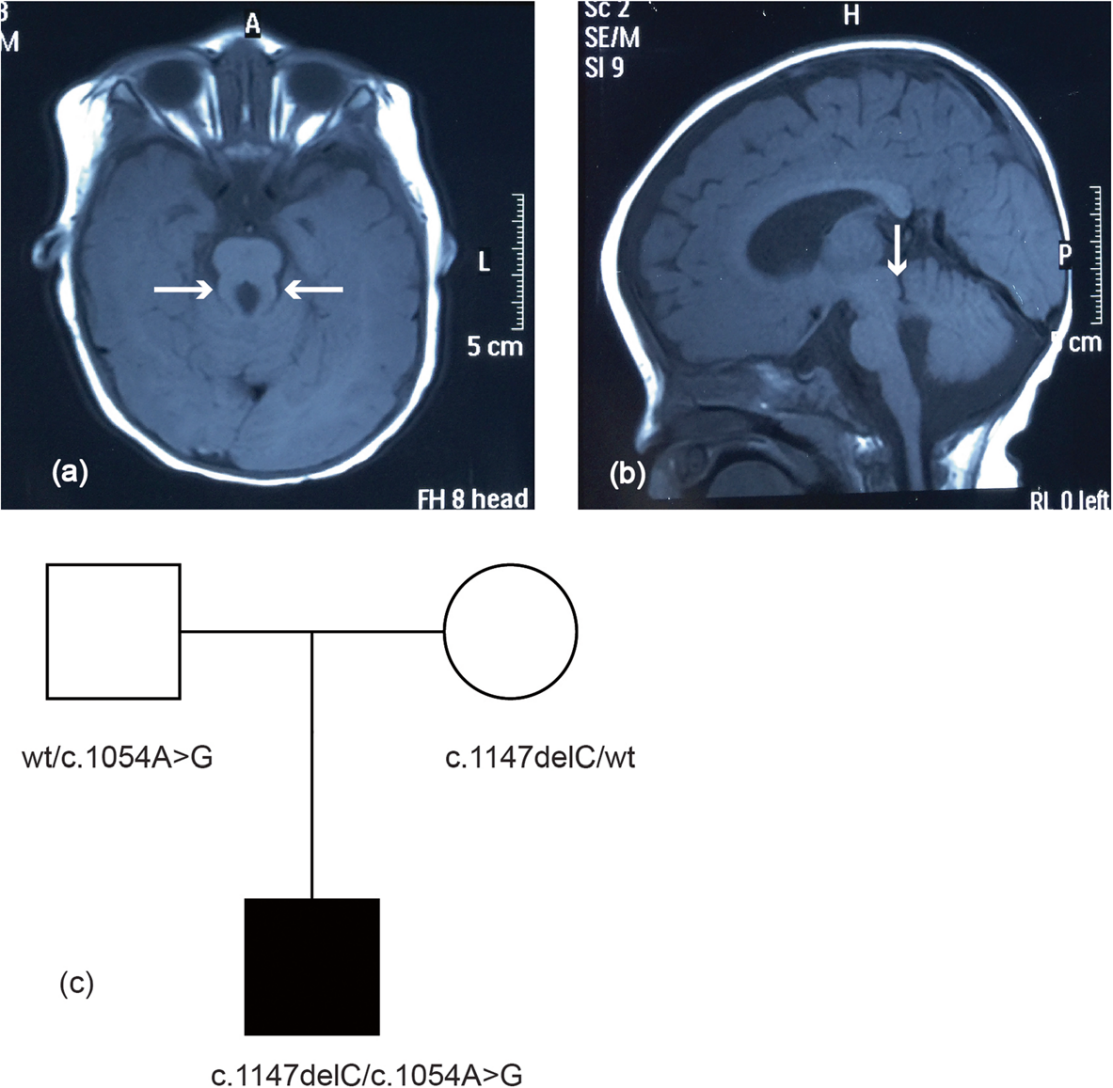
Brain magnetic resonance imaging (MRI) findings for the Joubert syndrome individual. Molar tooth sign with moderate cerebellar vermis hyoplasia, lengthening and thickening of superior cerebellar peduncles as well as superior cerebellar dysplasia as indicated by white arrows. **a** T2-weighted image. **b** T1-weighted image. **c** Pedigree of Joubert syndrome patient

A two-year-old boy was born to a nonconsanguineous couple by cesarean section with a birth weight of 3.42 kg, and the boy’s global development was delayed. He raised his head at 4 months, turned over at 8 months, sat all by himself at 10 months, crawled at 24 months, and stood with support at 28 months. The boy spoke with only monosyllables but had no other verbal communication skills. He had moderate intellectual disability evaluated by the Chinese Developmental Scale for children aged 0–6 years (WS/T 580–2017). The boy had a height of 95 cm (+ 2 SD), weight of 12.4 kg (0 SD), and occipitofrontal circumference of 53 cm (+ 3 SD) at 2 years. Physical examination showed frontal prominence, right eye esotropia, hypotonia and lower myodynamia. Ultrasonic examination showed normal liver, gallbladder, spleen, kidneys, ureter and bladder. Magnetic resonance imaging of his brain revealed unclear vermis of cerebellum, superior cerebellar peduncle thickening and lengthening (a clear molar tooth sign) as well as cerebellar hemispheres joining in the midline of brain and a smaller midbrain (Fig. 1). Thus, the boy was diagnosed with Joubert syndrome.

EDTA anticoagulant venous blood samples (2 mL) were obtained from all affected and unaffected family members. Genomic DNA was extracted from whole blood using the QIAamp® DNA Blood Mini Kit (QIAGEN, Germany) according to the manufacturer’s protocol, and whole exome sequencing was performed for the patient. An Agilent SureSelect Human All Exon V6 kit (Agilent Technologies Inc., USA) was used for preparation of the exome library using appromximately 3 μg of genomic DNA. The exome library was sequenced with a mean 100× coverage on an Illumina NovaSeq 6000 platform (Illumina Inc., USA). The average target coverage depth was 126× with > 97% of the bases covered at >20x, and the sensitivity was > 98%.

Raw data that passed quality control was aligned to the human reference genome (GRCh37/hg19), and only high-quality data (>Q30) was used for variant calling. Alignment to the human reference genome (GRCh37/hg19) and variant calling were performed using Burrows-Wheeler Aligner software (http://bio-bwa.sourceforge.net), followed by variant annotation by ANNOVAR [9] with the dbSNP147 databases (https://www.ncbi.nlm.nih.gov/SNP/), 1000G database (http://www.1000genomes.org/), ExAC database (http://exac.hms.harvard.edu/), HGMD (http://www.hgmd.cf.ac.uk/ac/index.php) and OMIM (https://www.ncbi.nlm.nih.gov/omim/).

The filtering criteria are shown in supplementary Table 1. The filtered variants were classified by the American College of Medical Genetics and Genomics (ACMG) standard to determine the pathogenic genes and variant sites. Conservation of different species was analyzed by MEGA6.0 software [10]. The candidate variants identified via whole exome sequencing were validated, and segregation analysis was performed by Sanger sequencing using the ABI3730xl Genetic Analyzer (Life Technologies, Carlsbad, CA) following the manufacturer’s protocol. The Sanger sequencing chromatogram was viewed by Chromas software and aligned to other reference sequences by SeqMan software. The PolyPhen-2 (http://genetics.bwh.harvard.edu/pph2/), PROVEAN (http://provean.jcvi.org/index.php) and Mutation Taster (http://www.mutationtaster.org/) online tools were used to predict the effect of candidate variants for protein function. The protein domains were analyzed by the SMART online service (http://smart.embl-heidelberg.de/).

**Fig. 2:**
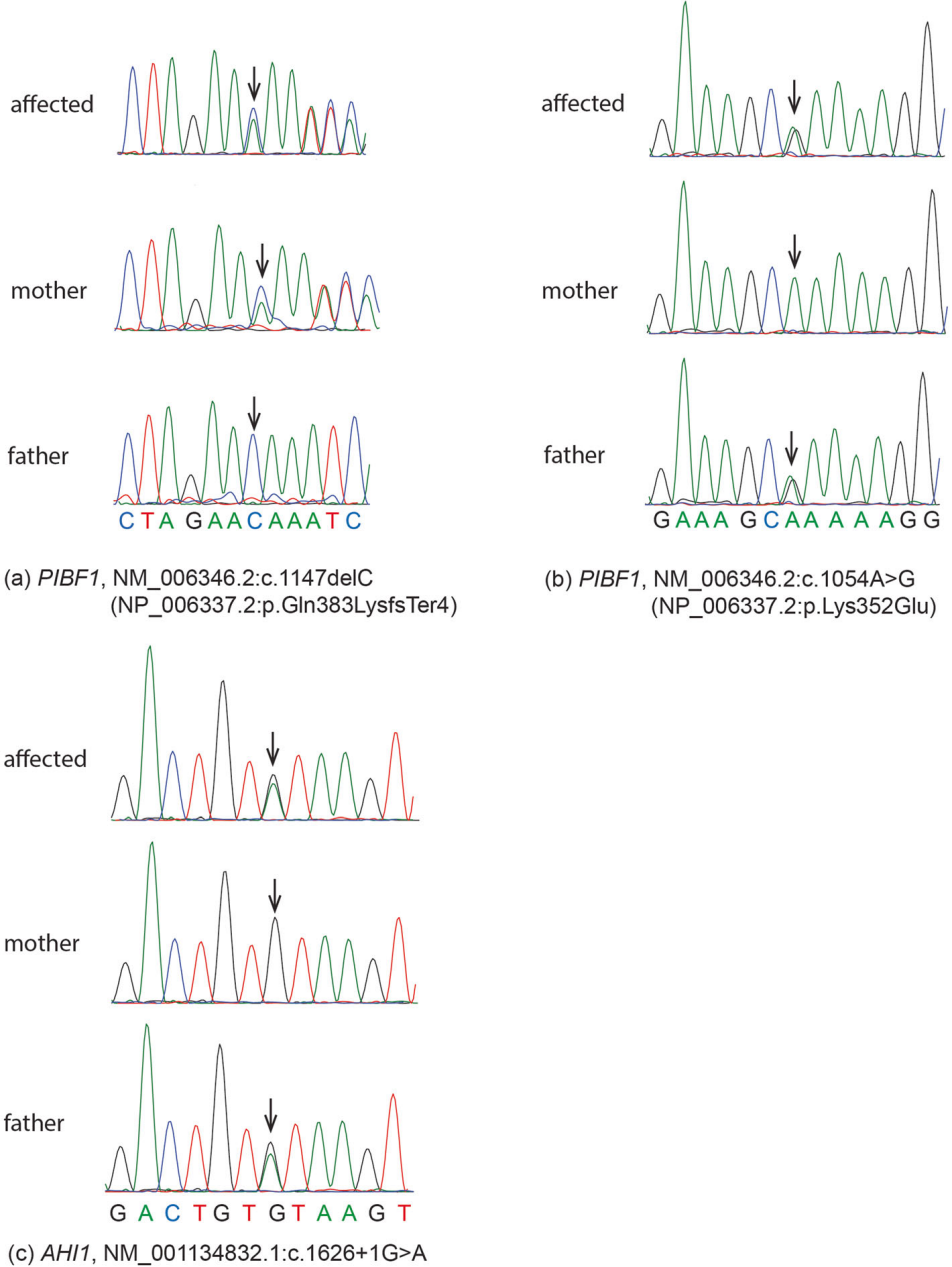
Sanger chromatograms. **a** c.1147delC variant in *PIBF1* in a heterozygous state in the proband (upper panel) and a heterozygous state in the mother (middle panel) and a wild-type homozygous state in the father (lower panel). **b** c.1054A > G variant in *PIBF1* in a heterozygous state in the proband (upper panel) and a wild-type homozygous state in the mother (middle panel) and a heterozygous state in the father (lower panel). **c** c.1626 + 1G > A variant in *AHI1* in a heterozygous state in the proband (upper panel) and a wild-type homozygous state in the mother (middle panel) and a heterozygous state in the father (lower panel)

Whole exome sequencing identified one variant (c.1626 + 1G > A) in *AHI1* (NM_001134832.1) and two variants (c.1147delC and c.1054A > G) in *PIBF1* (NM_006346.2) in a heterozygous state in the affected individual. Direct Sanger sequencing of the patient and parents showed that *AHI1* c.1626 + 1G > A was from his father (heterozygote) as his mother was wild-type for this variant. For *PIBF1*, only one heterozygote of these two variants was observed in his parents (c.1147delC in mother and c.1054A > G in father) (Fig. 2).

These two PIBFI variants were absent in the 1000 Genomes Project, ESP, gnomAD, Exome Variant Server (EVS) and Exome Aggregation Consortium (ExAC) datasets. The c.1147delC variant is located in exon 9 of *PIBF1*, leading to an amino acid change from glutamine to lysine at the position 383 of the *PIBF1* protein, which causes a frameshift, resulting a premature protein truncation (p.Gln383LysfsTer4). The c.1054A > G variant is located in exon 8 of *PIBF1*, and it is a missense variant in exon 8, leading to an amino acid change from lysine to glutamic acid at position 352 of the *PIBF1* protein (p.Lys352Glu). This variant was predicted to be probably damaging, deleterious and disease causing according to the protein predicted by PolyPhen-2 with a score of 0.962, PROVEAN with a score of − 2.833, and Mutation Taster with a score of > 0.999, respectively. According to the ACMG standards and guidelines, these *PIBF1* variants were classified as pathogenic (c.1147delC) and likely pathogenic (c.1054A > G) [11].

## Discussion and conclusions

We identified two novel pathogenic variants (NM_006346.2: c.1147delC and c.1054A > G) in *PIBF1* by whole exome sequencing of a Joubert syndrome individual. These two variants were present in heterozygous state in the affected child, which was consistent with the autosomal recessive inheritance mode. The c.1147delC variant was maternally inherited, and the c.1054A > G variant was paternally inherited.

*PIBF1* is located in chromosome 13q21-q22, contains 18 exons and spans more than 234 kb [12], and it encodes a predicted hydrophilic 757-amino acid alpha-helical protein [13], which is produced during pregnancy in response to progesterone [14]. Kim K. et al. reported that the *PIBF1* protein plays an important role in the formation of primary cilia [15]. *PIBF1* is a core component of the human centrosome and is crucial for the accumulation of centriolar satellites, eventually forming the primary cilia [15]. Depletion of *PIBF1* causes mitotic arrest, misaligned chromosomes and spindle pole fragmentation [15]. Exogenous expression of human wild-type *PIBF1* following siRNA knockdown rescues ciliogenesis in mIMCD3 cells [6]. A whole genome siRNA reverse genetics screen has identified recessive variants in *PIBF1* in seven individuals with Joubert syndrome [6].

**Fig. 3:**
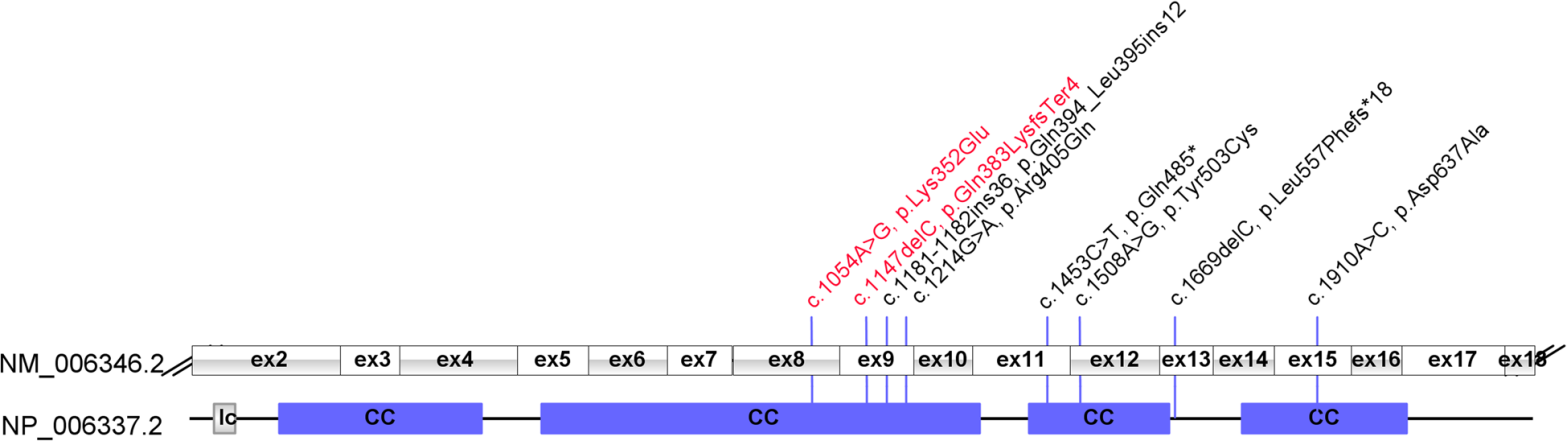
Pathogenic variants in the *PIBF1* gene and the protein structure. The novel variants of this study are marked in red. CC, coiled coil region; lc, low complexity region

Some variants of *PIBF1* have been identified to be associated with Joubert syndrome (Table 1) and all the variants are showed in Fig. 3. In this study, we found two novel variants in a Joubert syndrome individual. All Joubert syndrome individuals with *PIBF1* variants have a developmental delay and hypotonia, and most of these individuals have molar tooth sign and cerebellar vermis hypoplasia. Abnomal ocular movement was present in the affected boy of the present study and in a girl previously reported by Ott. T et al. Only Hutterite descent individuals shown the syndrome of ataxia (Table 1). Due to limited cases, the relationship of variant type and Joubert syndrome symptoms needs further investigation.

**Table 1:** Summary of clinical characteristics and PIBFI variants observed in Joubert syndrome patients

		current study	Wheway et al.^1^	Wheway et al.^1^	Hebbar M. et al.^2^	Ott T. et al.^3^
number of patients		1	1	6	1	1
gender		male	female	three female, three male	female	female
Origin		Chinese	NA	Canada Hutterite	Indian	German
clinical features	Developmental delay	+	+	all were +	+	+
	Hypotonia	+	+	all were +	+	+
	ocular movement abnormality	+	NA	all were NA	NA	+
	Ataxia	NA	+	all were +	NA	NA
	Cystic kidney disease	–	NA	all were NA	+	–
	Retinal degeneration	–	NA	all were NA	–	–
	Molar tooth sign	+	+	two patients was -, one was NA, and three was +	+	+
	Perisylvian polymicrogyria	–	–	all were -	+	+
	Hypoplasia of corpus callosum	–	–	all were -	+	–
	Cerebellar vermis hypoplasia	+	+	five patients were +, and one was NA	+	+
	Foramen magnum cephalocele	+	NA	four patients were -, one was NA, and one was +	–	–
variant details	mutation1	c.1147delC, p.Gln383LysfsTer4	c.1214G > A, p.Arg405Gln	c.1910A > C, p.Asp637Ala	c.1181_1182ins36, p.(Gln394_Leu395ins12)	c.1453C > T, p.Gln485∗
	mutation2	c.1054A > G, p.Lys352Glu	c.1669delC, p.Leu557Phefs*18	c.1910A > C, p.Asp637Ala	c.1181_1182ins36, p.(Gln394_Leu395ins12)	c.1508A > G, p.Tyr503Cys

*+* affected, *–* not affected, *NA* not available. 1. Wheway G, et.al. Nat Cell Biol. 2015;17(8):1074–1087. 2. Hebbar M, et al. J Hum Genet. 2018;63(8):935–939. 3. Ott T, et al. Front Physiol. 2019;10:134

A reported pathogenic variant (NM_001134832.1 c.1626 + 1G > A) in the *AHI1* gene was found in a heterozygous state in the patient and his father but as a wild-type in his mother, indicating that this variant did not segregate in the family. *AHI1* is located in chromosome 6q23.3, contains 33 exons and spans more than 213 kb [16]. Ferland R.J. et al. first reported the relationship between Joubert syndrome and *AHI1* variants [17]. Parisi, M. A. et al. reported that variants of *AHI1* cause both retinal dystrophy and renal cystic disease in Joubert syndrome patients [18]. To date, more than 10 variants associated with Joubert syndrome have been reported [17, 19–23]. *AHI1* c.1626 + 1G > A represents a G to A transition at the first base downstream of the 3′ end of exon 12 in *AHI1* as a splice variant. This variant was reported by Bachmann-Gagescu, R. et al., as a compound heterozygote combined with the c.2361G > T variant in *AHI1* to cause Joubert syndrome [19]. We screened all of the *AHI1* exons by Sanger sequencing for this Joubert syndrome patient and his parents, and we did not find another pathogenic or likely pathogenic variant in *AHI1,* except for c.1626 + 1G > A. Therefore, this variant was not the pathogenic cause for this child (data not shown).

Next generation sequencing has aided in the search for genetic variants of rare inherited diseases, such as Joubert syndrome. To date, however, Joubert syndrome can be treated but not cured. Thus, it is important to understand the underlying disease mechanism. Additional functional information is required to develop a treatment and comprehend the developmental regularity of the inherited diseases.

In conclusion, the present study identified two novel variants in *PIBF1* associated with Joubert syndrome and expanded the *PIBF1* pathogenic variant spectrum of Joubert syndrome. Further functional validation is necessary to clarify the pathogenic mechanism of the *PIBF1* gene in Joubert syndrome.

## Supplementary information

**Additional file 1.**

## Data Availability

The datasets generated and/or analyzed during the current study are available in the human reference 8 genome (GRCh37/hg19), NM_001134832.1, NM_006346.2.

## Abbreviations

*PIBF1*: Progesterone immunomodulatory binding factor 1
JBTS: Joubert syndrome
